# Improving Sperm Cryopreservation With Type III Antifreeze Protein: Proteomic Profiling of Cynomolgus Macaque (*Macaca fascicularis*) Sperm

**DOI:** 10.3389/fphys.2021.719346

**Published:** 2021-10-04

**Authors:** Bingbing Chen, Shengnan Wang, Briauna Marie Inglis, Hao Ding, Angbaji Suo, Shuai Qiu, Yanchao Duan, Xi Li, Shanshan Li, Wendell Q. Sun, Wei Si

**Affiliations:** ^1^Institute of Biothermal Science and Technology, School of Medical Instruments and Food Engineering, University of Shanghai for Science and Technology, Shanghai, China; ^2^State Key Laboratory of Primate Biomedical Research, Institute of Primate Translational Medicine, Kunming University of Science and Technology, Kunming, China

**Keywords:** cynomolgus macaques, sperm, cryopreservation, antifreeze protein III, proteomic profiling

## Abstract

Antifreeze protein III (AFP III) is used for the cryopreservation of germ cells in various animal species. However, the exact mechanism of its cryoprotection is largely unknown at the molecular level. In this study, we investigated the motility, acrosomal integrity, and mitochondrial membrane potential (MMP), as well as proteomic change, of cynomolgus macaque sperm after cryopreservation. Sperm motility, acrosomal integrity, and MMP were lower after cryopreservation (*p* < 0.001), but significant differences in sperm motility and MMP were observed between the AFP-treated sperm sample (Cryo+AFP) and the non-treated sample (Cryo–AFP) (*p* < 0.01). A total of 141 and 32 differentially expressed proteins were, respectively, identified in cynomolgus macaque sperm cryopreserved without and with 0.1 μg/ml AFP III compared with fresh sperm. These proteins were mainly involved in the mitochondrial production of reactive oxygen species (ROS), glutathione (GSH) synthesis, and cell apoptosis. The addition of AFP III in the sperm freezing medium resulted in significant stabilization of cellular molecular functions and/or biological processes in sperm, as illustrated by the extent of proteomic changes after freezing and thawing. According to the proteomic change of differentially expressed proteins, we hypothesized a novel molecular mechanism for cryoprotection that AFP III may reduce the release of cytochrome c and thereby reduce sperm apoptosis by modulating the production of ROS in mitochondria. The molecular mechanism that AFP III acts with sperm proteins for cellular protection against cryoinjuries needs further study.

## Introduction

Similarities between non-human primates and humans in physiology, genetics, and behavior make primates one of the widely used animal models in biomedical research. Primate models play essential roles in human disease research, drug development, and therapeutic strategy validation. The generation of primate models has been greatly accelerated as a result of the newly developed nuclease-based genome editing tools, such as CRISPR-Cas9 technology, and improvements of assisted reproduction technologies in primates (David, 2016). However, the cost, space, and labor required to maintain these models as living animals have created a huge burden to the biomedical community. As a result, there is an unprecedented need for optimal protocols for the maintenance of these models as cryopreserved germplasm (sperms and embryos). In combination with the established assisted reproductive technologies, such as artificial insemination, *in vitro* fertilization, and embryo transfer, in primate, cryopreservation of sperm and embryo provides efficient and cost-effective methods to safeguard primate models.

Sperm cryopreservation is an efficient method to safeguard primate models with a single mutation. However, the current protocol is not optimal. During the freezing and thawing process, sperms are exposed to several adverse events such as cold shock, intracellular ice formation, osmotic injury, pH change, oxidative stress and disruption of adenosine triphosphate (ATP) production. The stresses can inflict considerable cryo-damages on sperms (Parks and Graham, 1992; Muldrew and McGann, 1994; Gao and Critser, 2000; Johnson et al., 2000). Consequently, cryopreserved sperms show reduced motility, compromised acrosomal integrity, and low mitochondrial membrane potential (MMP), as well as impaired fertilizing capacity (Salamon and Maxwell, 1995; Gillan et al., 1997). Previously, we have successfully cryopreserved cynomolgus macaque sperm by optimizing a freezing protocol using a chemically defined medium (SpermCryo, All-round) designed for human sperm banking. However, the cryo-survival of sperm was still low after thawing compared with the traditional egg yolk-based extenders (Yan et al., 2016; Wang et al., 2019). Therefore, continued efforts are needed to optimize this freezing protocol.

Antifreeze proteins (AFPs) have thermal hysteresis ability that can inhibit ice recrystallization through binding to the surface of ice crystals to prevent the further propagation of ice crystals during freezing and thawing, especially during thawing, which can be fatal to cells (Kim et al., 2017). AFP III, the most widely used AFP, is a globular AFP with a highly stable structure formed by hydrogen bonds and hydrophobic interactions (Salvay et al., 2010). Recently, we demonstrated that supplementation of AFP III at 0.1 μg/ml to the clinical egg yolk-free medium (named “sperm freezing medium” or SFM) significantly improved the post-thaw motility and MMP (Wang et al., 2019). AFP III has also been shown to protect germ cells from a variety of animal species upon cryopreservation. However, the mechanism of its action remains largely unknown, besides the property of AFP to alter hydrogen bond dynamics in the aqueous solution (Salvay et al., 2010). Several studies have suggested that AFPs interact with membrane proteins, which can positively affect the survival of post-thaw sperms and oocytes (Lee et al., 2015; Saeed et al., 2020). The development of proteomic tools makes it possible to investigate the proteomic alteration of sperm after cryopreservation. A previous study indicated that there were 584 identified differentially expressed proteins in cryopreserved human sperm compared with the fresh sperm specimen (Li et al., 2019). A similar phenomenon about the qualitative changes of protein profiles after sperm cryopreservation has been reported in chicken, boar, ram and human sperm (Wang et al., 2013; Vilagran et al., 2014; Cheng et al., 2015; Bogle et al., 2017; Pini et al., 2018). However, there is no study about the effects of cryopreservation on non-human primate sperm proteome so far.

Therefore, the purpose of this research was to study the proteomic profiles of cynomolgus macaque sperm cryopreserved with SFM supplemented with AFP III (Wang et al., 2019) and identify the proteins affected by AFP III during the freezing and thawing process. This research has provided useful insight into the mechanism of cryoinjuries at a molecular level and illuminated the mechanisms by which antifreeze proteins may protect sperm during cryopreservation.

## Materials and Methods

### Animal and Ethics

All procedures of this study were approved by the Institutional Animal Care and Use Committee, Kunming University of Science and Technology (authorization code: LPBR201701001). A total of six healthy cynomolgus macaque males (age: from 7 to 12 years old), provided by the Yunnan Key Laboratory of Primate Biomedical Research (Kunming, China), were used as semen donors. Animals were kept in an animal room with 12:12 light–dark cycle at the room temperature of 18–26°C.

### Sperm Cryopreservation

Unless otherwise stated, all reagents were purchased from Millipore Sigma (St. Louis, MO, USA). The clinical egg yolk-free medium was purchased from ORIGIO (Knardrepvej, Malov, Denmark). Semen samples were collected via penile electro-ejaculation as described previously (Gould and Mann, 1988). An aliquot was taken from each semen sample which served as fresh control (referred as Fresh group). Then, each semen sample was divided into two equal parts, which were diluted by the TALP-Hepes medium containing 0.3% BSA (TH3) with 0.2 μg/ml AFP III (the Cryo+AFP group) or without AFP III (the Cryo–AFP group), respectively. Each sample was then further diluted dropwise with SFM at a ratio of 1:1 to reach a final concentration of 1 × 10^8^ sperm/ml and allowed to sit at room temperature for 10 min. The samples were then packed into 0.5 ml cryo-straws and sealed. The cryo-straws were cooled for 30 min by holding horizontally 0.5 cm above liquid nitrogen (LN_2_) and then directly plunged into LN_2_ for storage. All samples were thawed by vigorously shaking for 1 min in a 37°C water bath (Wang et al., 2019).

### Sperm Functional Evaluations

The motility of fresh and/or cryopreserved sperm was evaluated using a light microscope as previously described (Wang et al., 2019). Five microliters of sperm samples was placed on a pre-warmed Makler counting chamber (Sefi Medical Instruments, Haifa, Israel). At least 200 sperms per sample were counted under a light microscope under 200× magnification, and the percentage of motile sperm was determined.

The acrosomal integrity was examined by using the Alexa Flour-488-peanut agglutinin conjugate assay (Molecular Probes, Eugene, OR, USA) (Yang et al., 2011). Briefly, fresh and frozen-thawed samples were smeared on microscope slides. The slides were air-dried and then stained with 10 μg/ml Alexa Flour-488-peanut agglutinin solution at 37°C for 30 min under dark. After staining, the slides were washed by phosphate-buffered saline (PBS) and observed under fluorescence microscope at the excitation wavelength of 488 nm and emission wavelength of 530 nm. Sperm head with an even ample green fluorescence in acrosomal region was identified as sperm with intact acrosome, while sperm head with little or no green fluorescence in acrosome region was identified as sperm with damaged acrosome. At least 200 sperms per smear were evaluated.

Mitochondrial membrane potential was evaluated by using the JC-1 (5,5′,6,6′-tetrachloro-1,1′,3,3′-tetraethyl benzimidazole carbocyanine iodide, fluorescent cationic dye) assay kit according to the instruction of the manufacturer (Smiley et al., 1991). Each sample was incubated with JC-1 at 37°C for 20 min followed by two washes. Immediately after wash, all samples were analyzed under a fluorescence microscope with the 488 nm excitation wavelength. At least 200 sperms per sample were evaluated. Sperms showing orange or yellow fluorescence due to JC-1 aggregation in mitochondria were classified as sperms with intact mitochondria. On the contrary, sperms with damaged mitochondria have a green fluorescence since the JC-1 reagent was dispersed.

### Extraction of Sperm Proteins and LC-MS/MS Analysis

Sperm samples were washed with PBS twice by centrifugation at 200 g for 5 min. Proteomic analysis was performed according to the protocols provided by PTM Biolabs Inc. (Hangzhou, Zhejiang, China). In brief, samples from each of the three experimental groups in a lysis buffer were sonicated using a high-intensity ultrasonic processor three times on ice. The samples were then centrifuged at 12,000 *g* at 4°C for 10 min for supernatant collection. Protein concentration was measured with the BCA kit following the instructions of the manufacturer.

After dissolving in water containing 0.1% formic acid, the tryptic peptides were immediately loaded onto a homemade reversed-phase analytical column (25 cm length, 75 μm i.d.). At a constant flow rate of 300 nl/min on a nanoElute UHPLC system (Bruker Daltonics), peptides were separated with a gradient from 4 to 6% acetonitrile containing 0.1% formic acid in 2 min, 6–24% in 68 min, 24–32% in 14 min and climbing to 80% in 3 min, then holding at 80% for the last 3 min. The peptides were analyzed using mass spectrometry by the timsTOF Pro (Bruker Daltonics) with 1.60 kV electrospray voltage after being subjected to a Capillary source. Fragments and precursors were analyzed at an MS/MS scan range from 100 to 1,700 *m*/*z* on the TOF detector with parallel accumulation serial fragmentation (PASEF) mode of the timsTOF Pro. Precursors were selected for fragmentation with 10 PASEF-MS/MS scans and 0–5 charge states per cycle, and dynamic exclusion for 30 s.

### Selection of the Differentially Expressed Proteins

Proteins were identified by using the Maxquant search engine (v.1.6.6.0) to process the MS/MS data. The proteomics data from the mass spectrometry have been uploaded to the ProteomeXchange Consortium with the dataset identifier PXD024836 via the PRIDE partner repository. The identified differentially expressed proteins were screened by DESeq with *p* < 0.05 and *p*-adjusted < 0.1. We obtained three sets of differentially expressed protein spectra, namely, the Cryo–AFP vs. fresh sample, the Cryo+AFP vs. fresh sample, and Cryo+AFP vs. Cryo–AFP, through the pairwise comparison among three experimental groups.

### Bioinformatics Analysis and Statistical Analysis

Gene Ontology (GO) annotation was performed using the UniProt-GOA database (http://www.ebi.ac.uk/GOA/). Identified protein IDs were first converted to UniProt IDs and then mapped to GO IDs. Some identified proteins, unannotated by UniProt-GOA database, were annotated with the GO functional of the protein via the InterProScan software based on the protein sequence alignment method. The classification of differentially expressed proteins through GO was annotated on the basis of three categories: cellular component, molecular function, and biological process.

Differentially expressed proteins were annotated with the protein pathway using the Kyoto Encyclopedia of Genes and Genomes (KEGG) database. The KEGG database description of the protein was annotated by the KEGG online service tools KAAS, and the annotation result on the KEGG pathway database was mapped by using KEGG online service tools KEGG mapper.

All data were expressed as means ± SEM. The statistical analysis of sperm MMP, acrosomal integrity, and motility was performed using ANOVA and the Fisher's least-significance difference test (SPSS 16, SPSS, Chicago, IL, USA). A *p*-value < 0.05 is statistically significant.

## Results

### Effect of AFP III on the Motility, Acrosomal Integrity, and Mitochondrial Membrane Potential of Cynomolgus Macaque Sperm After Cryopreservation

**Figure 1:**
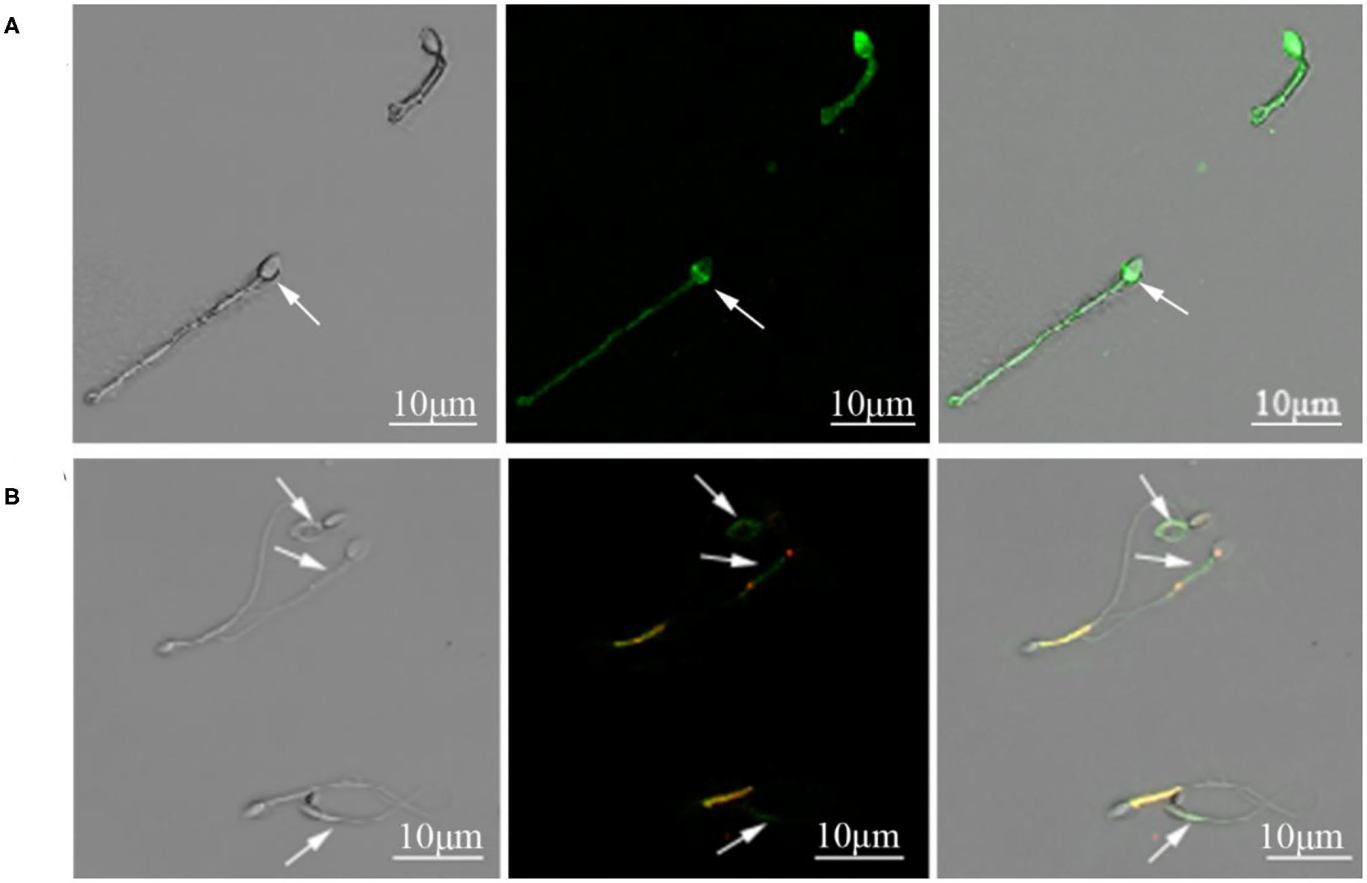
**(A)** Cynomolgus macaque sperm with intact and damaged acrosomes. The arrow indicated a sperm with damaged acrosome. **(B)** Sperm with intact and damaged mitochondria function. The arrows indicated sperm with compromised mitochondrial membrane potential.

The motility, acrosomal integrity, and MMP of fresh sperm samples (Fresh), frozen-thawed sperm cryopreserved with AFP III (Cryo+AFP), and frozen-thawed sperm cryopreserved without AFP III (Cryo–AFP) were shown in Table 1. The fluorescence images of sperm acrosomal integrity and MMP were shown in Figure 1. Compared with fresh sperm, cryopreservation significantly decreased the motility, acrosomal integrity, and MMP of sperm (*p* < 0.001). But sperm samples that were frozen with 0.1 μg/ml AFP III (Cryo+AFP) showed significantly higher post-thaw motility and MMP than those samples frozen without AFP III (Cryo–AFP) (*p* < 0.01). No difference in acrosomal integrity was observed in two cryopreserved groups (*p* > 0.05).

**Table 1:** The motility, acrosomal integrity, and mitochondrial membrane potential of fresh sperm and post-thaw sperm cryopreserved with or without AFP III (*n* = 6).

**Group**	**Sperm motility (%)**	**Acrosomal integrity (%)**	**Mitochondrial membrane potential (%)**
Fresh	83.7 ± 2.5%^a^	94.2 ± 1.2%^a^	80.3 ± 1.7%^a^
Cryo–AFP	31.8 ± 2.6%^c^	81.0 ± 2.0%^b^	51.8 ± 2.4%^c^
Cryo+AFP	44.3 ± 2.8%^b^	84.3 ± 2.1%^b^	65.2 ± 2.9%^b^

*Fresh, fresh sperm; Cryo–AFP, sperm cryopreserved without AFP III; Cryo+AFP, sperm cryopreserved with AFP III*.

*Significant difference was indicated by different superscripts in the same column (p < 0.05)*.

### Identification of Cynomolgus Macaque Sperm Proteins

**Figure 2:**
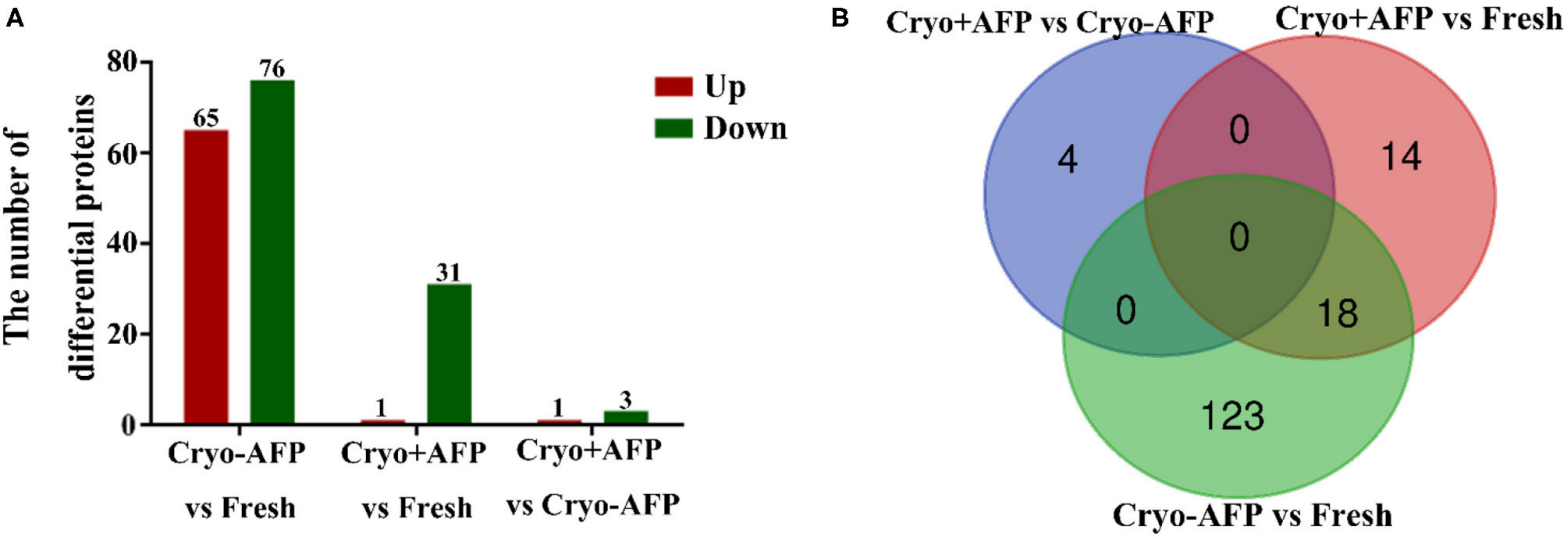
Identification of differentially expressed proteins in cynomolgus macaque sperm. **(A)** The number of differentially identified cynomolgus macaque sperm proteins between Fresh and Cryo–AFP, between Fresh and Cryo+AFP, and between Cryo–AFP and Cryo+AFP groups. The upregulated and downregulated proteins were represented by the red and green bars, respectively. **(B)** The differences in identified differentially expressed proteins as shown by the Venn diagram between Fresh, Cryo–AFP, and Cryo+AFP groups.

To determine whether there was somatic cell contamination in semen, the semen was stained with DAPI. In this study, we did not observe any somatic cell in the semen (Supplementary Figure 1). A total of 2,467 proteins were identified in the cynomolgus macaque sperms. Among them, 1,848 proteins were confidently quantified in six samples. Of special interest, 159 identified differential proteins were shared by sperm samples from the Fresh, Cryo–AFP, and Cryo+AFP groups (Figure 2; Supplementary Table 1). When compared with the fresh sperm sample, cryopreservation without AFP III (Cryo–AFP) resulted in the upregulation of 65 proteins and the downregulation of 76 proteins, whereas cryopreservation with AFP III (Cryo+AFP) only resulted in the upregulation of 1 protein and the downregulation of 31 proteins. This finding has clearly demonstrated that the supplementation of 0.1 μg/ml AFP III in the sperm freezing medium significantly helps the stabilization of cellular molecular functions or biological processes in sperms, as illustrated by the extent of proteomic changes after freezing and thawing. The further comparison between sperm samples cryopreserved with or without AFP III shows that the supplementation of APF III in the sperm freezing medium leads to the relative upregulation of 1 protein and the relative downregulation of 3 proteins as shown in Figure 2A. Based on the Venn diagram analysis of sperm differential proteins exhibited in Figure 2B, there were 18 differential proteins in common for both Cryo–AFP and Cryo+AFP groups when compared with the Fresh group, and 137 differential proteins different between Cryo–AFP vs. Fresh and Cryo+AFP vs. Fresh groups.

According to the UniProtKB database, 159 differential proteins with known roles in sperm function were used to further evaluate the effect of cryopreservation on cynomolgus macaque sperm function. The functions of selected sperm differential proteins including flagellated sperm motility, fertilization, mitochondrial function, heat/oxidative stress, apoptosis, metabolic process, enzymatic activity, immune response, ion binding, and others were listed in Supplementary Table 1.

### Gene Ontology Functional Analysis

**Figure 3:**
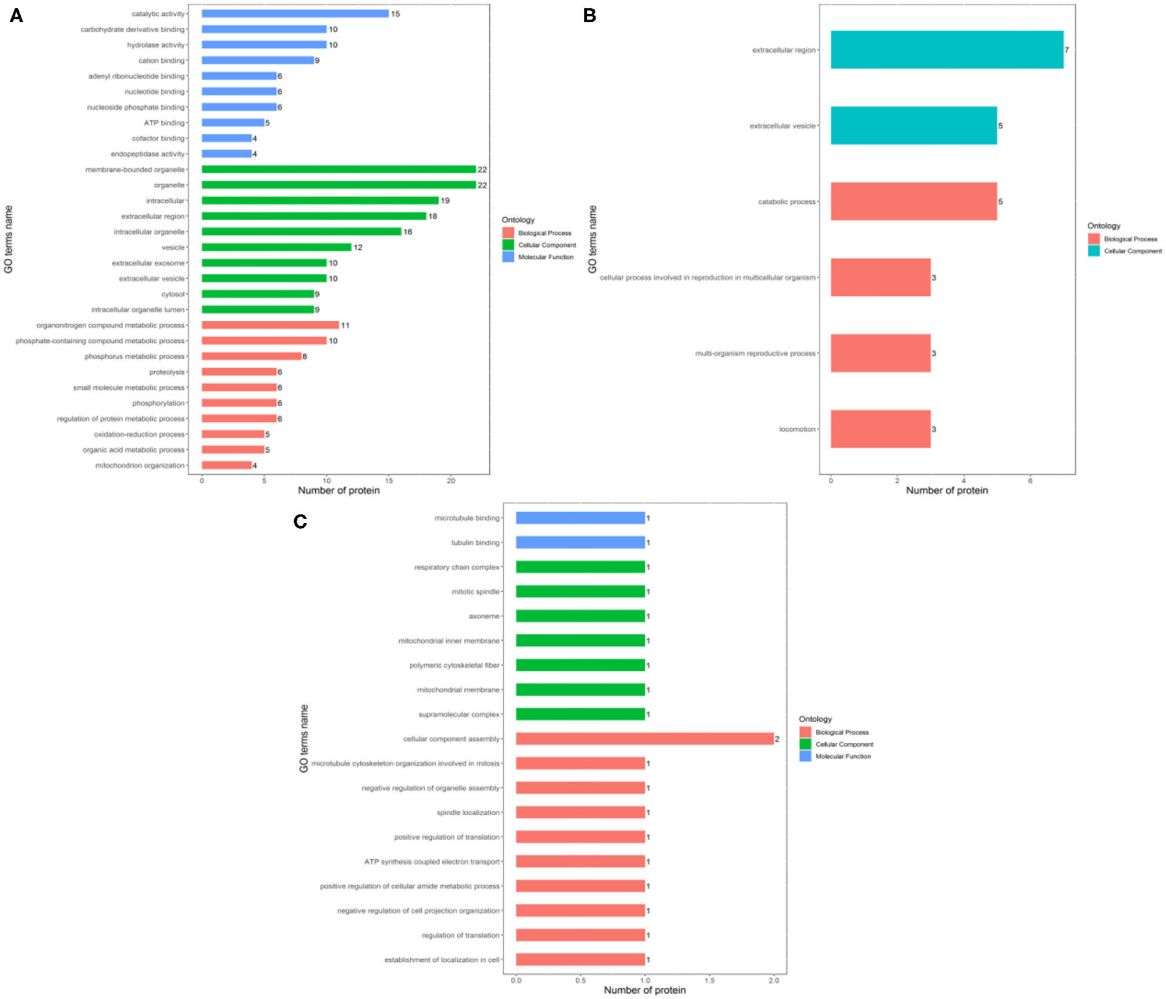
Biological process, cellular component, and molecular function of differentially expressed proteins. **(A)** between Fresh and Cryo–AFP groups; **(B)** between Fresh and Cryo+AFP groups; **(C)** between Cryo–AFP and Cryo+AFP groups. The value represents the number of differential proteins.

The identified differentially expressed proteins involving in biological processes, cellular components, and molecular function in GO terms between the Fresh control, Cryo–AFP and Cryo+AFP groups were shown in Figure 3. In Figure 3A, biological processes of differential proteins between Fresh and Cryo–AFP groups were primarily related to organonitrogen compound metabolic process (GO:1901564, 11 proteins), phosphorus metabolic process (GO:0006793, 8 proteins), phosphate-containing compound metabolic process (GO:0006796, 10 proteins), proteolysis (GO:0006508, 6 proteins), small molecule metabolic process (GO:0044281, 6 proteins), phosphorylation (GO:0016310, 6 proteins), regulation of protein metabolic process (GO:0051246, 6 proteins), oxidation–reduction process (GO:0055114, 5 proteins), organic acid metabolic process (GO:0006082, 5 proteins), and mitochondrion organization (GO:0007005, 4 proteins). Cellular components of differential proteins between Fresh and Cryo–AFP groups were enriched in the membrane-bound organelle (GO:0043227, 22 proteins), organelle (GO:0043226, 22 proteins), intracellular region (GO:0005622, 19 proteins), extracellular region (GO:0005576, 18 proteins), intracellular organelle (GO:0043229, 16 proteins), vesicle (GO:0031982, 12 proteins), extracellular exosome (GO:0070062, 10 proteins), extracellular vesicle (GO:1903561, 10 proteins), cytosol (GO:0005829, 9 proteins), and intracellular organelle lumen (GO:0070013, 9 proteins). The molecular function of differential proteins between Fresh and Cryo–AFP groups was enriched in catalytic activity (GO:0003824, 15 proteins), carbohydrate derivative binding (GO:0097367, 10 proteins), hydrolase activity (GO:0016787, 10 proteins), cation binding (GO:0043169, 9 proteins), adenyl ribonucleotide binding (GO:0032559, 6 proteins), nucleotide binding (GO:0000166, 6 proteins), nucleoside phosphate binding (GO:1901265, 6 proteins), ATP binding (GO:0005524, 5 proteins), cofactor binding (GO:0048037, 4 proteins), and endopeptidase activity (GO:0004175, 4 proteins).

In Figure 3B, biological processes of the identified differential proteins in the GO terms were basically catabolic process (GO:0009056, 5 proteins), cellular process involved in reproduction in multicellular organism (GO:0022412, 3 proteins), multi-organism reproductive process (GO:0044703, 3 proteins), and locomotion (GO:0040011, 3 proteins) between Fresh and Cryo+AFP groups. The identified differential proteins-associated cellular localizations were enriched in the extracellular region (GO:0005576, 7 proteins) and extracellular vesicle (GO:1903561, 5 proteins) between Fresh and Cryo+AFP groups.

In Figure 3C, biological processes of the identified differential proteins between Cryo–AFP and Cryo+AFP groups were involved in the cellular component assembly (GO:0022607, 2 proteins), microtubule cytoskeleton organization involved in mitosis (GO:1902850, 1 proteins), negative regulation of organelle assembly (GO:1902116, 1 protein), spindle localization (GO:0051653, 1 protein), positive regulation of translation (GO:0045727, 1 protein), ATP synthesis-coupled electron transport (GO:0042773, 1 protein), negative regulation of cell projection organization (GO:0031345, 1 protein), positive regulation of cellular amide metabolic process (GO:0034250, 1 protein), regulation of translation (GO:0006417, 1 protein), and establishment of localization in the cell (GO:0051649, 1 protein). The identified differential proteins-associated cellular localizations were enriched in respiratory chain complex (GO:0098803, 1 protein), mitotic spindle (GO:0072686, 1 protein), axoneme (GO:0005930,1 protein), mitochondrial inner membrane (GO:0005743,1 proteins), polymeric cytoskeletal fiber (GO:0099513, 1 protein), mitochondrial membrane (GO:0031966, 1 protein), and supramolecular complex (GO:0099080, 1 protein). The molecular function of the identified differential proteins between Cryo–AFP and Cryo+AFP groups is enriched in microtubule binding (GO:0008017, 1 protein) and tubulin binding (GO:0015631, 1 protein).

### Enrichment-Based Clustering

**Figure 4:**
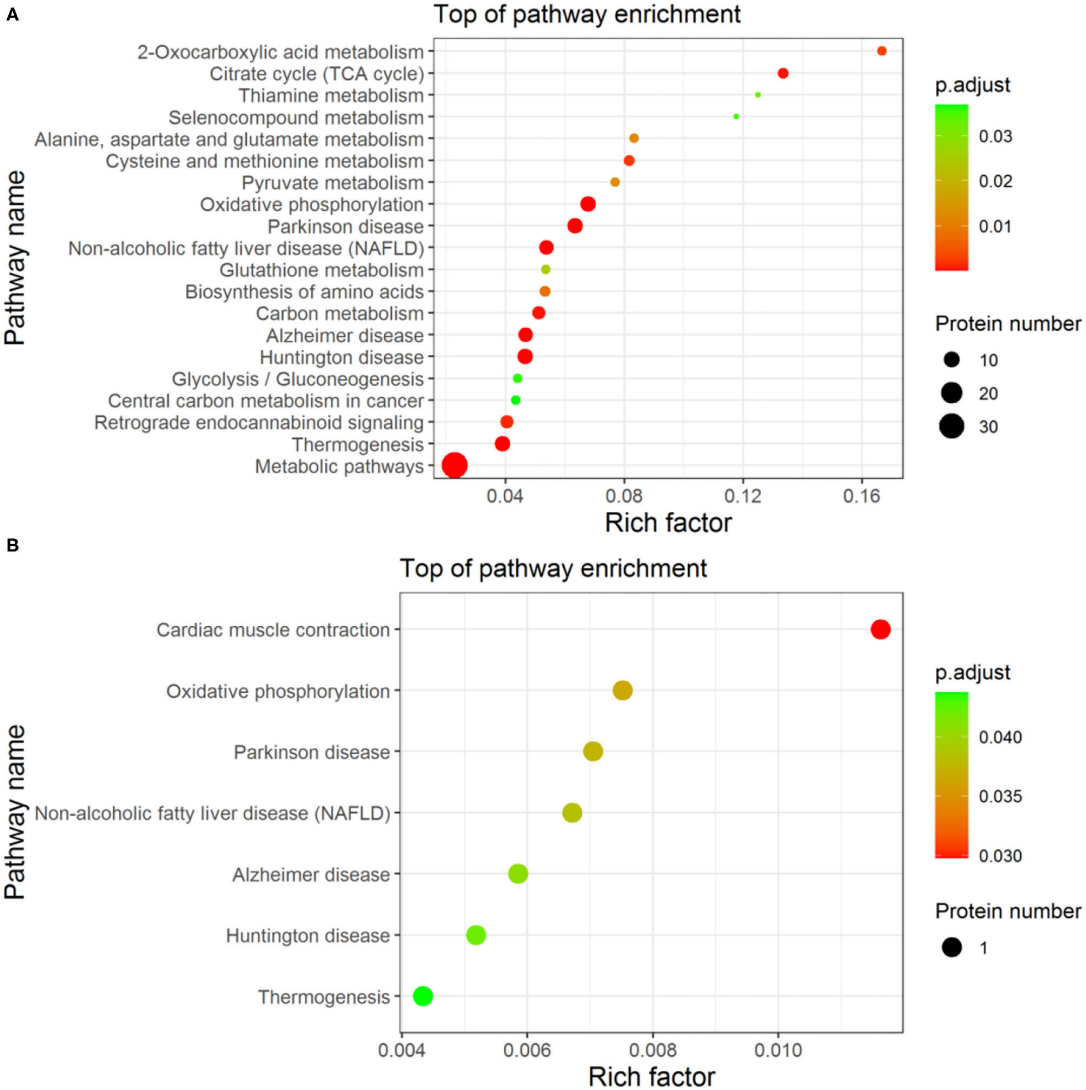
The enrichment analysis-based clustering of the KEGG pathway. **(A)** between Fresh and Cryo–AFP groups; **(B)** between Cryo–AFP and Cryo+AFP groups.

The enrichment analysis-based clustering of the identified differential proteins from Fresh, Cryo–AFP, and Cryo+AFP groups was exhibited in Figure 4. The statistical analysis of the KEGG pathway of the identified differential proteins is enriched in 2-oxocarboxylic acid metabolism, alanine, aspartate, and glutamate metabolism, citrate cycle (TCA cycle), selenocompound metabolism, thiamine metabolism, oxidative phosphorylation, Parkinson's disease, pyruvate metabolism, non-alcoholic fatty liver disease (NAFLD), cysteine and methionine metabolism, glutathione metabolism, biosynthesis of amino acids, carbon metabolism, Alzheimer disease, Huntington disease, glycolysis/gluconeogenesis, central carbon metabolism in cancer, retrograde endocannabinoid signaling, thermogenesis and metabolic pathways between the Fresh and Cryo–AFP groups; and thermogenesis, Huntington disease, Alzheimer disease, non-alcoholic fatty liver disease (NAFLD), Parkinson's disease, oxidative phosphorylation, and cardiac muscle contraction between the Cryo–AFP and Cryo+AFP groups. Differential proteins between the Fresh and Cryo+AFP groups were not enriched into any pathways.

## Discussion

In this study, we investigated the effect of AFP III on cynomolgus macaque sperm cryopreservation. The results demonstrated that sperm motility, acrosomal integrity, and MMP were decreased significantly after the freezing and thawing process. The addition of 0.1 μg/ml of AFP III into the sperm freezing medium reduced sperm damage and improved the motility and MMP of sperm after cryopreservation. Proteomic analysis showed that cryopreservation resulted in the alteration of proteomic patterns in cynomolgus macaque sperm, especially those proteins associated with flagellated sperm motility, fertilization, mitochondrial function, heat/oxidative stress, apoptosis, metabolic process, enzymatic activity, immune response, ion binding, etc. The addition of AFP III in the sperm freezing medium protects sperm during cryopreservation by stabilizing mitochondrial function, reducing ROS production, and protecting sperm from apoptosis as evidenced by relative differential proteins by proteomic analysis.

A total of 159 differentially expressed proteins involving in sperm functions were identified after sperm cryopreservation among Fresh, Cryo–AFP, and Cryo+AFP groups. Compared with the Fresh group, 18 proteins were downregulated in both Cryo–AFP and Cryo+AFP groups. Those proteins were associated with fertilization, apoptosis, metabolic process and enzymatic activity, which likely pinpoints the main cause of the decreased sperm motility and fertility after cryopreservation (Xu et al., 2020). For example, the levels of zona pellucida binding protein (G7P1S4, ZPBP), zona pellucida binding protein 2 (A0A2K5U0C3, ZPBP2), acrosin-binding protein (A0A2K5UCT2), and phosphatidylethanolamine-binding protein 4 (A0A2K5X0P4, PEBP4) were decreased in both Cryo–AFP and Cryo+AFP groups. The loss of ZPBP, ZPBP2, and acrosin-binding protein of cryopreserved sperm could result in the deficiency to penetrate zona pellucida and initiation of acrosome reaction (Lin et al., 2007). It has been reported that the compromised acrosome reaction contributes to the subfertility of the acrosin-binding protein-deficient mice (Nagashima et al., 2019). Furthermore, the inhibition of endogenous PEBP4 expression in MCF-7 cells was found to be associated with the decreased expression of anti-apoptotic proteins such as BclXL and Bcl-2 and the increased expression of the pro-apoptotic proteins p21CIP/WAF, p53, and Bax (Wang et al., 2005). The decreased level of PEBP4 in cryopreserved sperm is likely correlated with the tendency of sperm apoptosis.

Also compared with the Fresh group, there were additional 137 differential proteins that were found either in the Cryo–AFP group (123 proteins) or in the Cryo+AFP group (14 proteins). Again, those proteins were largely associated with flagellated sperm motility, fertilization, mitochondrial function, heat/oxidative stress, apoptosis, metabolic process, enzymatic activity, immune response, and ion binding. Clearly, the changes in the proteomic patterns after freezing and thawing would have a profound negative impact on sperm motility and fertility, and the presence of AFP III in the sperm freezing medium could reduce this impact. The proteomic analysis shows that the levels of proteins related to ROS generation in mitochondrial complex, such as NDUFS8, NDUFB6, CYC1 and OCIAD1, were increased in the Cryo–AFP group when compared with Fresh group, but their levels were relatively unchanged in the Cryo+AFP group, indicating that AFP III could reduce the ROS production in mitochondrial complex I upon cryopreservation. NADH dehydrogenase (ubiquinone) iron–sulfur protein 8 (A0A2K5U649, NDUFS8) is a mitochondrial Fe–S protein in complex I (a major contributor of ROS generation) located in the inner membrane of mitochondria and participates in the electron transport chain. The upregulation of NDUFS8 increases the mitochondrial ROS production (Cheng et al., 2013). NADH dehydrogenase (ubiquinone) 1 beta subcomplex subunit 6 (A0A2K5VNL0, NDUFB6) is an accessory subunit of the multi-subunit NADH in complex I associated with ROS production, ATP generation, and cell apoptosis in the mitochondrial inner membrane. The increased expression of NDUFB6 is accompanied by the increase of ROS (Wang et al., 2020). It has been proposed that the increased ROS production during cryopreservation could lead to DNA modification, lipid peroxidation, and/or protein damages, which in turn induces cell apoptosis because of the mitochondrial cytochrome c release and the disruption of cellular homeostasis (Stokman et al., 2017). Cytochrome c 1 (A0A2K5WMQ0, CYC1), an electron carrier between complex III and complex IV in mitochondrial respiration chain located outside the mitochondrial inner membrane (Xia et al., 2002; Zhao et al., 2020), binds to APAF-1 after releasing into cytoplasm, activates pro-caspase 9, and triggers an enzymatic cascade leading to cell death (Santucci et al., 2019). Complex I activity is correlated negatively with OCIA domain-containing protein (A0A2K5VJ27, OCIAD1) expression (Shetty et al., 2018). We hypothesized that the presence of AFP III in the sperm freezing medium can reduce mitochondrial ROS production and sperm apoptosis, which may explain the higher sperm motility and MMP in the Cryo+AFP group than the Cryo–AFP group.

Another major group of differential proteins between the Fresh and Cryo–AFP and Fresh and Cryo+AFP groups is the GSH synthesis-related proteins, including gamma-ECS, lactoylglutathione lyase, and carbonyl reductase 1. GSH ensures the normal function of cell apoptosis by maintaining redox homeostasis and resisting oxidant aggression. This study detects the downregulation of gamma-ECS (A0A2K5UV75, GCLC), lactoylglutathione lyase (Q4R5F2, GLO1), and carbonyl reductase 1 (Q8MI29, CBR1) in the Cryo–AFP group when compared with the Fresh group, but not in the Cryo+AFP group. GCLC participates in the first rate-limiting reaction in GSH synthesis and feedback inhibited by GSH itself to the regulation of cellular GSH concentration (Griffith and Meister, 1979). The reduced GCLC expression increased the methylglyoxal-induced pheochromocytoma cells apoptosis (Kimura et al., 2009). By converting the spontaneously formed MGO-GSH hemithioacetal to the thioester S-d-lactoylglutathione, GLO1 acts as the rate-limiting enzyme in the primary detoxification step. GSH concentration is directly proportional to GLO1 activity. The impaired GLO1 activity is associated with the decreased GSH concentration (Sousa Silva et al., 2013; Nigro et al., 2017) and with the induced apoptosis of acute myeloid leukemia cells (Chen et al., 2015). CBR1 inactivates cellular membrane-derived lipid aldehydes to protect cells from oxidative stress and cell apoptosis. The study has found that lipid peroxidation products and oxidative stress markers were significantly lower in cells overexpressing CBR1. In contrast, the level of oxidative stress protein expressed by CBR1 knockout cells was increased (Kwon et al., 2019). Therefore, our results also suggest that AFP III could reduce ROS production by protecting the GSH synthesis-related proteins during freezing and thawing.

Furthermore, the downregulation of a non-specific serine/threonine protein kinase (A0A2K5UYV4, STK39) and a stress-induced protein (Q4R8N7, STIP1) and the upregulation of a non-specific T-complex protein 1 subunit (Q4R5J0, CCT8) were observed only in the Cryo–AFP group, but not in Cryo+AFP group after sperm cryopreservation in this study. The suppression of STK39 significantly induces cell apoptosis in 786-0 and ACHN cells. STK39 knockdown reduces the anti-apoptosis protein Bcl-2 and increases the apoptosis-promoting protein Bax (Zhao et al., 2018). The downregulation of STIP1 increases cell apoptosis of glioma cells (Yin et al., 2019). The upregulation of CCT8 was reported to be connected to the neuronal apoptosis in adult rats with traumatic brain injury (Robles et al., 2015). Again, these findings appear to suggest a role of AFP III in preventing the apoptosis of sperm after freezing and thawing.

Of particular interest are the four differential proteins between the Cryo–AFP and Cryo+AFP groups (Figure 2B). The upregulated one was the ubiquinol-cytochrome-c reductase complex assembly factor 1 (A0A2K5WI39, UQCC1), whereas the downregulated ones were the cytochrome c oxidase subunit 3 (C3W4Z1, COX3), the transmembrane helical component TEX51 (A0A2K5X818), and the microtubule-associated protein (A0A2K5UU31, MAP4). Those proteins are associated with mitochondrial electron and redox function, membrane assemblies, and cytoskeleton organization. UQCC1, the complex III assembly factors, participates in the cytochrome b biogenesis (Tucker et al., 2013). COX3, the catalytic core of cytochrome c oxidase, accepts electrons from cytochrome c and subsequently transfers them to molecular oxygen to generate water (Little et al., 2018). Some previous studies have shown that cryopreservation alters the composition of sperm proteins (Wang et al., 2013; Vilagran et al., 2014; Cheng et al., 2015; Bogle et al., 2017; Pini et al., 2018; Li et al., 2019). During the freezing and thawing process, the loss of intracellular components due to damaged membranes may contribute to the considerable loss of some sperm proteins, whereas the increases of some sperm proteins can be due to secondary or tertiary structure transformations or degradation of proteins (Bogle et al., 2017). This study demonstrates that sperm cryopreservation changes the levels of some proteins in both the Cryo–AFP and Cryo+AFP groups, which results in a decrease in the number of differential proteins between the two groups. For example, the levels of 18 decreased proteins in both Cryo–AFP and Cryo+AFP groups were similar (Supplementary Table 1).

Previous studies demonstrate that AFP III reduces the damage of cellular structures by ice crystallization during freezing and thawing (Salvay et al., 2010; Saeed et al., 2020). AFP III can also stabilize the cell membrane, thereby reducing the sublethal damage during sperm cryopreservation (Robles et al., 2019). This study has extended our understanding of the cryoprotective role of AFP III by demonstrating that AFP III can maintain mitochondrial function and reduce ROS production and sperm apoptosis during the freezing and thawing process. The finding is consistent with the findings that the addition of AFP III successfully improved sperm motility and mitochondrial membrane potential. Sperm cryopreservation increases the production of ROS, such as superoxide and hydrogen peroxide, in many species including human and rhesus macaque (McCarthy and Meyers, 2011; Lee et al., 2015; Saeed et al., 2020). In another unpublished paper, we demonstrated that sperm cryopreservation could induce ROS production in cynomolgus monkey. AFP III has been proved to have an anti-peroxide effect in sperm cryopreservation. For example, human sperm cryopreservation with AFP III improved motility and total antioxidant capacity (TAC) levels. Meanwhile, AFP III decreased the percentage of DNA fragmentation and ROS level (Kim et al., 2017). Furthermore, ROS level was decreased in mouse oocytes vitrified with AFP III (Salvay et al., 2010). Another study also demonstrated that reduced glutathione/oxidative glutathione (GSH/GSSG) and total antioxidant capacity (TAC) were higher in bull semen cryopreservation with AFP III compared with the control group (Jang et al., 2020). In our study, the results also suggested that AFP III could protect the proteins associated with ROS production in mitochondria and GSH synthesis during sperm freezing and thawing. The proteomic evidence in this study is consistent with previous studies, suggesting the anti-peroxidative effect of AFP III. Therefore, we hypothesized that AFP III may reduce cryo-damages of sperm structures, as well as the release of cytochrome c, thereby reducing sperm apoptosis by suppressing the mitochondrial ROS production and enhancing the antioxidative function.

## Conclusion

The addition of AFP III in the sperm freezing medium protects sperm during cryopreservation. The proteomic analysis has identified 159 proteins with known functions that are susceptible to sperm cryopreservation. The addition of 0.1 μg/ml of AFP III in the sperm freezing medium can change the outcome, significantly reducing the number of differential proteins in cryopreserved sperm. According to the biological processes and molecular functions of differentially expressed proteins, we proposed a new molecular mechanism for AFP III cryoprotection that AFP III may reduce sperm apoptosis by reducing the release of cytochrome c and the mitochondrial ROS production. The findings provide insight into the AFP III cryoprotection mechanism and a useful hint for a new strategy of developing and optimizing sperm cryopreservation. However, the mechanism of AFP III modulating the proteomic profiles upon cryopreservation needs to be studied further.

## Data Availability Statement

The datasets presented in this study can be found in online repositories. The names of the repository/repositories and accession number(s) can be found in the article/Supplementary Material.

## Ethics Statement

The animal study was reviewed and approved by the Yunnan Key Laboratory of Primate Biomedical Research (Kunming, China) and all procedures were approved by the Institutional Animal Care and Use Committee, Kunming University of Science and Technology (authorization code: LPBR201701001), and were executed according to the Guide for Care and Use of Laboratory Animals (Commission on Life Sciences, the National Research Council, Washington, D.C.).

## Author Contributions

WS and BC conceptualized the study. BC, HD, and AS contributed to the formal analysis. BC, WQS, and WS contributed to the writing (review and editing). BC, SW, and BI contributed to the investigation. SQ, YD, Chen Adar, Ido Braslavsky, XL, and SL contributed to the resources. All authors have read and agreed to the published version of the manuscript.

## Funding

This research was supported by grants from the National Key Research and Development Program of China (Grant Number: 2018YFA0801403) and Yunnan Fundamental Research Projects (Grant Number: 2018FA020).

## Conflict of Interest

The authors declare that the research was conducted in the absence of any commercial or financial relationships that could be construed as a potential conflict of interest.

## Publisher's Note

All claims expressed in this article are solely those of the authors and do not necessarily represent those of their affiliated organizations, or those of the publisher, the editors and the reviewers. Any product that may be evaluated in this article, or claim that may be made by its manufacturer, is not guaranteed or endorsed by the publisher.

## Supplementary Material

Supplementary Figure 1 Cynomolgus macaque semen was stained with DAPI. No somatic cells were observed in the semen.
