# Supplementary figures and images for: Improving Sperm Cryopreservation With Type III Antifreeze Protein: Proteomic Profiling of Cynomolgus Macaque (Macaca fascicularis) Sperm
Source: Front Physiol. 2021 Oct 4;12:719346. doi: 10.3389/fphys.2021.719346 (PMC8521148; doi:10.3389/fphys.2021.719346)

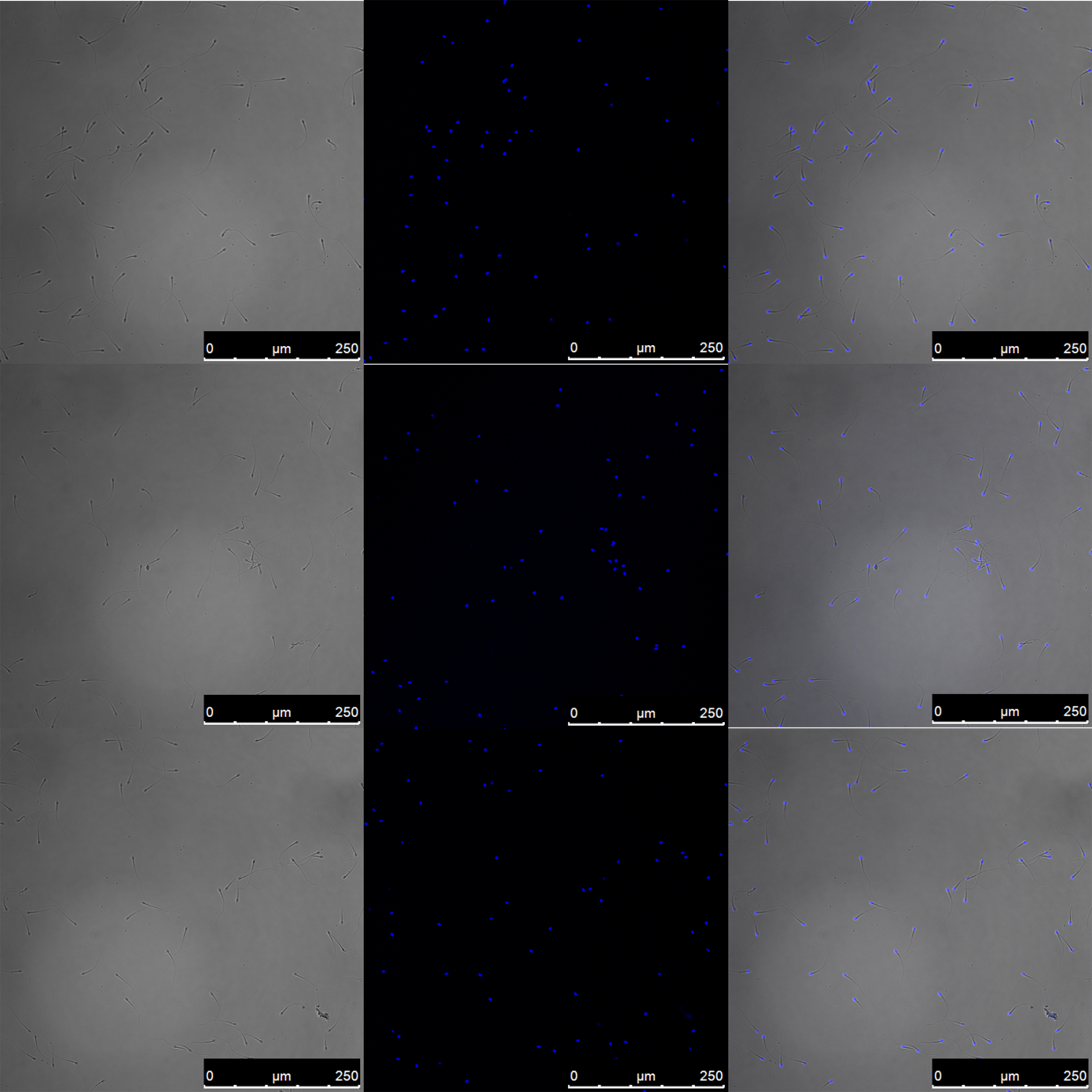

Supplement: Supplementary Figure 1 — Cynomolgus macaque semen was stained with DAPI. No somatic cells were observed in the semen. [file Image_1.PNG]
