# Supplementary material for: Improving Sperm Cryopreservation With Type III Antifreeze Protein: Proteomic Profiling of Cynomolgus Macaque (Macaca fascicularis) Sperm
Source: Front Physiol. 2021 Oct 4;12:719346. doi: 10.3389/fphys.2021.719346 (PMC8521148; doi:10.3389/fphys.2021.719346)
Supplement: Supplementary file 2 [file Table_1.docx]

# Supplementary Tables

**Table S1.** 159 differential proteins of the cynomolgus macaque sperm in three groups

| **Biological processes** | **Protein accession** | **Protein name** | **Gene name** | **Cryo-AFP/ Fresh Ratio** | **Cryo+AFP /Fresh Ratio** | **Cryo+AFP/ Cryo-AFP Ratio** |
| --- | --- | --- | --- | --- | --- | --- |
| Flagellated sperm motility | A0A2K5V5S9 | Uncharacterized protein | TCTEX1D2 | 18.27↑ |  |  |
|  | A0A2K5TWR3 | Tubulin beta chain |  | 4.10↑ |  |  |
|  | G7P706 | Profilin | PFN3 | 2.62↑ |  |  |
|  | Q4R6C5 | Ropporin-1 (Rhophilin-associated protein 1) | ROPN1 | 2.16↑ |  |  |
|  | A0A2K5WKM3 | Primary cilia formation | PIFO | 1.90↑ |  |  |
|  | A0A2K5UM23 | Dynein light chain roadblock | DYNLRB2 | 1.37↑ |  |  |
|  | Q4R3A0 | Tubulin polymerization-promoting protein family member 2 | TPPP2 | 1.32↓ |  |  |
|  | A0A2K5W6F0 | FERM domain-containing protein | EZR |  | 3.18↓ |  |
| Fertilization | G7P1F3 | Peptidase S1 domain-containing protein |  | 5.01↓ |  |  |
|  | A0A2K5UC72 | Complement component 4 binding protein alpha | C4BPA | 2.66↓ | 2.52↓ |  |
|  | A0A2K5U0C3 | Zona pellucida binding protein 2 | ZPBP2 | 2.19↓ | 2.39↓ |  |
|  | G7P1S4 | Zona pellucida binding protein | ZPBP | 2.03↓ | 1.68↓ |  |
|  | A0A2K5UCT2 | Acrosin-binding protein |  | 1.61↓ | 1.56↓ |  |
|  | A0A2K5VW50 | Testis expressed 37 | TEX37 | 1.74↑ |  |  |
|  | A0A2K5WH00 | Acrosin | ACR | 1.51↓ |  |  |
|  | G7PU48 | Lysozyme like 6 | LYZL6 | 1.38↓ |  |  |
|  | G7Q2M1 | Sperm acrosome associated 5 |  | 1.68↓ |  |  |
| Mitochondrial function | A0A2K5W9K0 | Uncharacterized protein | PAM16 | 18.31↑ |  |  |
|  | A0A2K5VAT8 | Coiled-coil domain containing 51 | CCDC51 | 18.02↑ |  |  |
|  | A0A2K5X1V0 | Stomatin like 2 | STOML2 | 3.18↑ |  |  |
|  | G7P1E9 | Single stranded DNA binding protein 1 | SSBP1 | 2.93↑ |  |  |
|  | A0A2K5WMQ0 | Cytochrome c1 | CYC1 | 2.33↑ |  |  |
|  | G8F4P2 | Complex (NADH dehydrogenase [ubiquinone] 1 alpha subcomplex subunit 7) |  | 2.27↓ |  |  |
|  | Q8SPH5 | Cytochrome b-c1 complex subunit 6, | UQCRH | 2.09↑ |  |  |
|  | A0A2K5WBY0 | Complex I-B14.7 |  | 2.01↑ |  |  |
|  | A0A2K5VNL0 | NADH: ubiquinone oxidoreductase subunit B6 | NDUFB6 | 1.80↑ |  |  |
|  | A0A2K5TLU9 | ATP synthase membrane subunit f (ATP synthase subunit f, mitochondrial) |  | 1.69↑ |  |  |
|  | A0A2K5TS24 | NADH dehydrogenase [ubiquinone] 1 beta subcomplex subunit 8 |  | 1.37↑ |  |  |
|  | A0A2K5W0Z0 | Prohibitin | PHB | 1.23↑ |  |  |
|  | Q8SPH6 | ATP synthase-coupling factor 6 | ATP5PF/ATP5J |  | 2.71↑ |  |
|  | C3W4Z1 | Cytochrome c oxidase subunit 3 | COX3 |  |  | 2.81↓ |
|  | A0A2K5WI39 | Ubiquinol-cytochrome-c reductase complex assembly factor 1 | UQCC1 |  |  | 18.42↑ |
| Heat/oxidative stress | I7G9X7 | Macaca fascicularis brain cDNA clone: QorA-13308, similar to human hypothetical protein MGC4248 (MGC4248) |  | 18.22↑ |  |  |
|  | A0A2K5UTB4 | DnaJ heat shock protein family (Hsp40) member A4 | DNAJA4 | 1.51↓ |  |  |
|  | P28714 | Epididymal secretory glutathione peroxidase | GPX5 |  | 3.35↓ |  |
| Apoptosis | G7PMI3 | Deoxyguanosine kinase | DGUOK | 2.66↑ |  |  |
|  | Q4R5F2 | Lactoylglutathione lyase | GLO1 | 2.44↓ |  |  |
|  | A0A2K5VHS2 | Serine/threonine kinase 11 | STK11 | 2.42↓ |  |  |
|  | A0A2K5U286 | Guided entry of tail-anchored proteins factor 4 | GET4 | 1.88↓ |  |  |
|  | A0A2K5X0P4 | Phosphatidylethanolamine binding protein 4 | PEBP4 | 1.69↓ | 1.71↓ |  |
|  | G7PQ90 | NADH:ubiquinone oxidoreductase core subunit S3 | NDUFS3 | 1.59↑ |  |  |
| Metabolic process | A0A2K5WA68 | Aspartate transaminase | GOT1L1 | 18.15↑ |  |  |
|  | A0A2K5U017 | Alpha-galactosidase | NAGA | 3.19↓ |  |  |
|  | A0A2K5U2A2 | L-lactate dehydrogenase |  | 2.29↓ |  |  |
|  | Q95LV1 | Beta-galactosidase-1-like protein | GLB1L | 2.28↓ | 1.89↓ |  |
|  | A0A2K5W815 | Protein kinase cAMP-dependent type I regulatory subunit beta | PRKAR1B | 2.20↓ | 2.13↓ |  |
|  | A0A2K5WN81 | CN hydrolase domain-containing protein |  | 2.18↓ |  |  |
|  | A0A2K5WZV8 | Phospholipase B-like | PLBD2 | 2.03↓ | 1.79↓ |  |
|  | G7P9W2 | Thiamine-triphosphatase |  | 1.97↓ |  |  |
|  | A0A2K5TW74 | Aminotran_1_2 domain-containing protein |  | 1.90↓ |  |  |
|  | A0A2K5UEA6 | Isochorismatase domain-containing protein 2 | ISOC2 | 1.90↑ |  |  |
|  | I7GHF9 | Macaca fascicularis brain cDNA clone: QorA-11170, similar to human UBX domain containing 1 (UBXD1) |  | 1.87↑ |  |  |
|  | A0A2K5V0D6 | Adenylate kinase isoenzyme 1 (AK 1) | AK1 | 1.76↑ |  |  |
|  | A0A2K5U998 | Alpha-L-fucosidase | FUCA1 | 1.69↓ |  |  |
|  | A0A2K5WBE9 | 6-phosphogluconate dehydrogenase, decarboxylating |  | 1.64↓ |  |  |
|  | A0A2K5WRH7 | Isocitrate dehydrogenase [NAD] subunit, mitochondrial | IDH3G | 1.60↑ |  |  |
|  | Q8MI29 | Carbonyl reductase [NADPH] 1 | CBR1 | 1.59↓ |  |  |
|  | A0A2K5UNA0 | Acetyltransferase component of pyruvate dehydrogenase complex |  | 1.51↑ |  |  |
|  | A0A2K5WJA5 | Isocitrate dehydrogenase [NADP] | IDH1 | 1.50↓ |  |  |
|  | G7P2A3 | Phosphoglycerate mutase | PGAM2 | 1.47↓ |  |  |
|  | A0A2K5VWX2 | 6-phosphofructo-2-kinase (Fructose-2,6-bisphosphatase) |  | 1.46↓ |  |  |
|  | Q60HC8 | Ubiquitin carboxyl-terminal hydrolase isozyme L1 | UCHL1 | 1.44↓ |  |  |
|  | A0A2K5WSR3 | PAW domain-containing protein |  | 1.34↓ |  |  |
|  | A0A2K5WD70 | Beta-hexosaminidase |  |  | 3.29↓ |  |
| Enzymatic activity | A0A2K5UYV4 | Non-specific serine/threonine protein kinase | STK39 | 18.71↓ |  |  |
|  | A0A2K5V2T0 | Uroporphyrinogen decarboxylase | UROD | 18.14↑ |  |  |
|  | A0A2K5W4I2 | Fumarylacetoacetate hydrolase domain containing 1 | FAHD1 | 5.10↓ |  |  |
|  | A0A2K5U7X2 | Peptidase S1 domain-containing protein |  | 2.57↑ |  |  |
|  | A0A2K5WT20 | Superoxide dismutase |  | 2.47↑ |  |  |
|  | A0A2K5UTG9 | Prolylcarboxypeptidase | PRCP | 2.35↓ | 2.05↓ |  |
|  | A0A2K5UZG9 | Peptidase S1 domain-containing protein | PRSS8 | 2.18↑ |  |  |
|  | A0A2K5WN73 | Peptidase S1 domain-containing protein |  | 2.15↓ |  |  |
|  | A0A2K5VGG5 | N-acetylglucosamine kinase | NAGK | 2.15↓ |  |  |
|  | A0A2K5X359 | Adenylosuccinate lyase |  | 2.04↓ |  |  |
|  | A0A2K5U7K2 | Ubiquitin carboxyl-terminal hydrolase |  | 1.96↓ | 1.90↓ |  |
|  | A0A2K5UV75 | Glutamate-cysteine ligase catalytic subunit | GCLC | 1.86↓ |  |  |
|  | A0A2K5WVY7 | Membrane metalloendopeptidase like 1 | MMEL1 | 1.86↓ |  |  |
|  | A0A2K5VT48 | Dihydrolipoamide acetyltransferase component of pyruvate dehydrogenase complex | DBT | 1.83↑ |  |  |
|  | A0A2K5VA83 | Atlastin GTPase 3 | ATL3 | 1.81↑ |  |  |
|  | A0A2K5TTV9 | Protein phosphatase, Mg2+/Mn2+ dependent 1A | PPM1A | 1.79↓ |  |  |
|  | A0A2K5TV58 | Amine oxidase | IL4I1 | 1.78↓ | 1.71↓ |  |
|  | A0A2K5TTC7 | Ribonuclease A family member 9 (inactive) | RNASE9 | 1.96↓ | 1.99↓ |  |
|  | A0A2K5VR26 | Carboxypeptidase A5 | CPA5 | 2.13↓ | 1.92↓ |  |
|  | A0A2K5WQA9 | Pseudouridine 5'-phosphatase | PUDP | 2.08↓ | 1.92↓ |  |
|  | A0A2K5VFA1 | Amino_oxidase domain-containing protein |  | 1.59↓ |  |  |
|  | A0A2K5URF9 | Phospholipase A2 inhibitor and LY6/PLAUR domain containing | PINLYP | 1.55↓ |  |  |
|  | A0A2K5WVS2 | Peptidyl-prolyl cis-trans isomerase | PPIL6 | 1.47↑ |  |  |
|  | A0A2K5X069 | Glutamine-dependent NAD(+) synthetase (NAD(+) synthase [glutamine-hydrolyzing]) |  | 1.44↓ |  |  |
|  | A0A2K5U649 | Complex I-23kD (NADH dehydrogenase [ubiquinone] iron-sulfur protein 8, mitochondrial) (NADH-ubiquinone oxidoreductase 23 kDa subunit) | NDUFS8 | 1.41↑ |  |  |
|  | A0A2K5WKT2 | Glutaminyl-peptide cyclotransferase (EC 2.3.2.5) | QPCT |  | 3.34↓ |  |
|  | A0A2K5U2A3 | TGc domain-containing protein | TGM4 |  | 3.14↓ |  |
|  | A0A2K5VFN5 | FABP domain-containing protein |  |  | 1.69↓ |  |
|  | A0A2K5WBJ0 | N(4)-(beta-N-acetylglucosaminyl)-L-asparaginase | AGA | 2.10↓ |  |  |
| Immune response | A0A2K5VH33 | Cochlin | COCH | 1.98↓ |  |  |
|  | Q8SQ46 | CD59 glycoprotein | CD59 | 1.77↓ |  |  |
|  | A0A2K5ULB9 | Lipocln_cytosolic_FA-bd_dom domain-containing protein | LCN2 |  | 3.75↓ |  |
|  | A0A2K5U3C2 | Secretory leukocyte peptidase inhibitor | SLPI |  | 3.48↓ |  |
|  | A0A2K5WA30 | SASA domain-containing protein |  |  | 2.70↓ |  |
| ion binding | A0A2K5TVL5 | Uncharacterized protein | PITPNB | 18.59↓ |  |  |
|  | A0A2K5UH95 | Ferritin |  | 18.17↑ |  |  |
|  | A0A2K5VIL4 | 2Fe-2S ferredoxin-type domain-containing protein |  | 18.11↑ |  |  |
|  | A0A2K5UE84 | Calcium-activated neutral proteinase small subunit |  | 4.34↓ |  |  |
|  | Q4R4X6 | Ras-related protein Rab-2A | RAB2A | 1.78↑ |  |  |
|  | A0A2K5UX30 | Glycerol-3-phosphate dehydrogenase, mitochondrial | GPD2 | 1.67↑ |  |  |
|  | A0A2K5TV74 | Aconitase 1 | ACO1 | 1.43↓ |  |  |
|  | A0A2K5V0Y0 | Deoxyhypusine hydroxylase | DOHH | 1.29↓ |  |  |
|  | Q95JS1 | 1-phosphatidylinositol 4,5-bisphosphate phosphodiesterase zeta-1 | PLCZ1 |  | 4.45↓ |  |
| Other | A0A2K5WY59 | Prostate-specific antigen | KLK3 | 18.97↓ |  |  |
|  | A0A2K5W207 | Peptidase_M24 domain-containing protein |  | 18.63↑ |  |  |
|  | A0A2K5UQB7 | Casein kinase II subunit beta (CK II beta) |  | 18.55↑ |  |  |
|  | A0A2K5U9P8 | Fibronectin type III and SPRY domain containing 1 like | FSD1L | 18.40↑ |  |  |
|  | A0A2K5V463 | Uncharacterized protein | MLF1 | 18.35↑ |  |  |
|  | G7PR10 | Leucine-rich repeat-containing protein 51 (Protein LRTOMT1) |  | 18.29↑ |  |  |
|  | A0A2K5VAY1 | ATP-binding cassette sub-family A member 3-like |  | 18.23↑ |  |  |
|  | G8F3G6 | Sodium/hydrogen exchanger 9B1 | SLC9B1 | 18.23↑ |  |  |
|  | A0A2K5US16 | Spermatogenesis-associated protein 17 | SPATA17 | 18.19↑ |  |  |
|  | A0A2K5UBM2 | Chromosome 5 open reading frame 49 | C5orf49 | 18.18↑ |  |  |
|  | Q4R8E6 | Proline-rich protein 30 | PRR30 | 18.17↑ |  |  |
|  | A0A2K5WSP3 | PA28_beta domain-containing protein |  | 18.13↑ |  |  |
|  | A0A2K5UET5 | Methionine--tRNA ligase, cytoplasmic (Methionyl-tRNA synthetase) | MARS1 | 18.11↑ |  |  |
|  | A0A2K5VI41 | Osteoclast stimulating factor 1 | OSTF1 | 18.03↑ |  |  |
|  | A0A2K5VAS3 | Uncharacterized protein |  | 18.01↑ |  |  |
|  | G7PBY1 | J domain-containing protein | DNAJC5B | 18.01↑ |  |  |
|  | A0A2K5VX54 | AB hydrolase-1 domain-containing protein | ABHD11 | 17.99↑ |  |  |
|  | A0A2K5UMU0 | Thioredoxin domain-containing protein 17 | TXNDC17 | 17.75↑ |  |  |
|  | A0A2K5VCK0 | Cystatin C | CST3 | 5.48↓ |  |  |
|  | Q4R323 | Protein FAM71C | FAM71C | 5.17↑ |  |  |
|  | A0A2K5V873 | IQ motif containing F5 | IQCF5 | 3.51↑ |  |  |
|  | A0A2K5VJ27 | OCIA domain containing 1 | OCIAD1 | 3.35↑ |  |  |
|  | A0A2K5V8A7 | Dynein light chain Tctex-type 1 | DYNLT1 | 3.07↑ |  |  |
|  | A0A2K5URM8 | Signal regulatory protein delta | SIRPD | 2.92↓ | 2.77↓ |  |
|  | A0A2K5WWJ1 | Chromosome 10 open reading frame 120 | C10orf120 | 2.88↓ |  |  |
|  | A0A2K5VN07 | LRRC37AB_C domain-containing protein |  | 2.80↓ | 2.64↓ |  |
|  | A0A2K5V7P8 | Uncharacterized protein |  | 2.74↓ |  |  |
|  | A0A2K5VAP6 | SPATA31 subfamily D member 1 | SPATA31D1 | 2.33↓ | 1.98↓ |  |
|  | A0A2K5W4D9 | ShKT domain-containing protein |  | 2.29↓ |  |  |
|  | A0A2K5TJK6 | SH3 domain-containing protein 21 | SH3D21 | 2.15↑ |  |  |
|  | A0A2K5TPH5 | ACB domain-containing protein | DBI | 2.03↓ |  |  |
|  | A0A2K5UKU1 | LRRC37AB_C domain-containing protein |  | 2.03↓ | 1.71↓ |  |
|  | G8F314 | Transmembrane protein 89 | TMEM89 | 1.97↑ |  |  |
|  | Q4R8N7 | Stress-induced-phosphoprotein 1 | STIP1 | 1.94↓ |  |  |
|  | A0A2K5WRI0 | Tetraspanin 16 | TSPAN16 | 1.84↑ |  |  |
|  | G7NYK6 | Non-specific serine/threonine protein kinase | OXSR1 | 1.83↓ |  |  |
|  | A0A2K5U7V9 | Uncharacterized protein |  | 1.79↓ |  |  |
|  | G7PB22 | Leucine rich repeat containing 74A | LRRC74A | 1.73↑ |  |  |
|  | A0A2K5X7M7 | Uncharacterized protein |  | 1.70↓ |  |  |
|  | A0A2K5WCZ3 | Coiled-coil domain-containing protein 116 | CCDC116 | 1.70↑ |  |  |
|  | A5LFW8 | Uncharacterized protein C19orf71 homolog | QtsA-14935 | 1.61↑ |  |  |
|  | A0A2K5V3I5 | Uncharacterized protein |  | 1.60↓ |  |  |
|  | A0A2K5VJT1 | Uncharacterized protein |  | 1.56↓ |  |  |
|  | Q4R5J0 | T-complex protein 1 subunit theta (TCP-1-theta) (CCT-theta) | CCT8 | 1.55↑ |  |  |
|  | A0A2K5TUG6 | Bleomycin hydrolase | BLMH | 1.36↓ |  |  |
|  | A0A2K5V7M0 | N-ethylmaleimide sensitive factor, vesicle fusing ATPase | NSF | 1.34↓ |  |  |
|  | Q8HXX7 | Rab GDP dissociation inhibitor alpha | GDI1 | 1.32↓ |  |  |
|  | A0A2K5WN01 | Uncharacterized protein |  |  | 2.18↓ |  |
|  | A0A2K5X818 | Uncharacterized LOC107126877 | TEX51 |  |  | 18.77↓ |
|  | A0A2K5UU31 | Microtubule-associated protein | MAP4 |  |  | 18.76↓ |
|  | A0A2K5X5G1 | Glypican 2 | GPC2 |  | 4.81↓ |  |
|  | A0A2K5VNQ8 | Uncharacterized protein |  |  | 3.75↓ |  |

↑ represents upregulation of protein, ↓ represents downregulation of protein.
